# Genetic dissection of thousand-seed weight in linseed (*Linum usitatissimum* L.) using multi-locus genome-wide association study

**DOI:** 10.3389/fpls.2023.1166728

**Published:** 2023-06-02

**Authors:** Ankit Saroha, Sunil S. Gomashe, Vikender Kaur, Deepa Pal, Shraddha Ujjainwal, J. Aravind, Mamta Singh, S. Rajkumar, Kuldeep Singh, Ashok Kumar, Dhammaprakash Pandhari Wankhede

**Affiliations:** ^1^ Division of Genomic Resources, Indian Council of Agricultural Research (ICAR)-National Bureau of Plant Genetic Resources, New Delhi, India; ^2^ ICAR-National Bureau of Plant Genetic Resources, Regional Station Akola, Maharashtra, India; ^3^ Division of Germplasm Evaluation, ICAR-National Bureau of Plant Genetic Resources, New Delhi, India; ^4^ Division of Germplasm Conservation, ICAR-National Bureau of Plant Genetic Resources, New Delhi, India; ^5^ ICAR-National Bureau of Plant Genetic Resources, New Delhi, India

**Keywords:** linseed, flaxseed, seed weight, genome-wide association studies, quantitative trait nucleotides, candidate genes

## Abstract

Flaxseed/linseed is an important oilseed crop having applications in the food, nutraceutical, and paint industry. Seed weight is one of the most crucial determinants of seed yield in linseed. Here, quantitative trait nucleotides (QTNs) associated with thousand-seed weight (TSW) have been identified using multi-locus genome-wide association study (ML-GWAS). Field evaluation was carried out in five environments in multi-year-location trials. SNP genotyping information of the AM panel of 131 accessions comprising 68,925 SNPs was employed for ML-GWAS. From the six ML-GWAS methods employed, five methods helped identify a total of 84 unique significant QTNs for TSW. QTNs identified in ≥ 2 methods/environments were designated as stable QTNs. Accordingly, 30 stable QTNs have been identified for TSW accounting up to 38.65% trait variation. Alleles with positive effect on trait were analyzed for 12 strong QTNs with *r* ^2^ ≥ 10.00%, which showed significant association of specific alleles with higher trait value in three or more environments. A total of 23 candidate genes have been identified for TSW, which included *B3 domain-containing transcription factor*, *SUMO-activating enzyme*, *protein SCARECROW*, *shaggy-related protein kinase/BIN2*, *ANTIAUXIN-RESISTANT 3*, *RING-type E3 ubiquitin transferase E4*, *auxin response factors*, *WRKY transcription factor*, and *CBS domain-containing protein*. *In silico* expression analysis of candidate genes was performed to validate their possible role in different stages of seed development process. The results from this study provide significant insight and elevate our understanding on genetic architecture of TSW trait in linseed.

## Introduction

Linseed/flaxseed (*Linum usitatissimum* L.) is an important crop in several countries across the world and has myriad of food, nutritional, and industrial applications. It is a self-pollinated diploid crop species (2*n* = 2*x* = 30) with genome size of ~368–373 Mb (Ragupathy et al., 2011; Wang et al., 2012). Linseed is considered to be originated in the Middle East or Indian regions (Vavilov, 1951; Green et al., 2008). There are two morphotypes of *L. usitatissimum*, “flax type” and “linseed type” (Diederichsen and Ulrich, 2009; Kaur et al., 2017). The linseed type is short statured, branched with high number of capsules bearing larger seeds.

Although, linseed is among the earliest domesticated crop and has been under cultivation since ancient times, in recent years, there is a surge of renewed interest and popularity largely due to the health benefits it offers. It is one of the richest plant-based sources of omega-3 fatty acid, alpha linolenic acid, and has an impressive nutritional profile with high amounts of proteins, dietary fibers, vitamin B1, and lignans, especially secoisolariciresinol diglucoside (SDG) (Yadav et al., 2022). The nutritional value of linseed extends to a wide range of health benefits in cardiovascular disease, cancer, neurological disorder, and other lifestyle related diseases/conditions such as diabetes, atherosclerosis, hypertension, and obesity (Pan et al., 2009; Katare et al., 2012; Goyal et al., 2014; Kajla et al., 2015; Mohammadi-Sartang et al., 2017). Accordingly, the higher demand has led to an upward trend in the global production of linseed in the last decade (FAOSTAT, 2022; Yadav et al., 2022). India, which ranks seventh in the world in terms of linseed production, however, has productivity substantially lower (0.543 tonne/ha) than the world average (1.053 tonne/ha) (Kaur et al., 2017).

Thousand-seed weight (TSW) (1,000 seed weight) is one of the major components of seed yield in linseed. Seed weight is widely recognized as a complex trait controlled by many genetic and environmental factors (Kraft et al., 1963; Mian et al., 1996). The synergistic growth of embryo, endosperm, and ovule contributes to seed development, wherein several signaling pathways and regulatory networks coupled with spatiotemporal disposition of an array of phytohormones play critical roles (Cao et al., 2020). Signaling pathways such as G-protein signaling, IKU (HAIKU), ubiquitin-proteosome pathway, and phytohormones especially auxins and associated factors have a role in the regulation of seed development and seed weight (Locascio et al., 2014; Cao et al., 2020). Several genes encoding Indole-3-acetic acid (IAA)–glucose hydrolase, GLYCOGEN SYNTHASE KINASE 3/SHAGGY-like, GRAS transcription factor, AUXIN RESPONSE FACTOR (ARF), WRKY transcription factor, and cytochrome P450 enzyme have been identified crucial in seed development and seed weight in other plants (Ishimaru et al., 2013; Chen et al., 2015; Liu et al., 2015a; Zhu et al., 2015; Ge et al., 2016; Hirano et al., 2017; Hu et al., 2018; Cao et al., 2020).

In linseed, a few research groups have attempted genetic dissection of seed weight trait using biparental linkage mapping and association studies in natural population and identified 44 unique quantitative trait loci (QTLs) (Soto-Cerda et al., 2014; Kumar et al., 2015; Xie et al., 2018a; Xie et al., 2018b; Guo et al., 2020; You and Cloutier, 2020). Among the earliest studies to understand the genetic architecture of TSW in linseed, Soto-Cerda et al. (2014) using association mapping identified five SSR markers associated with TSW, explaining 30% of the trait variation in the Canadian flax core collection. Kumar et al. (2015) using biparental mapping population (RILs) developed an SSR-SNP genetic map and identified a minor QTL for TSW encompassing important genes encoding leucine-rich receptor-like protein kinase, cytochrome P450, WRKY, and GRAS family transcription factors. Further using genome-wide association study (GWAS) and GWAS combined with LD heatmap in flax core and cultivated accessions, important candidate genes for TSW have been identified, namely, *SPX & EXS domain-containing protein* (*PHO1*), *ubiquitin-proteosome pathway genes*, *RING/U box protein*, *cytochrome P450*, *Auxin canalization (AC)*, and *26S proteosome regulatory subunit (RPN)* (Xie et al., 2018a; Guo et al., 2020).

As known from plethora of studies, seed weight is considered a complex trait governed by several genes. In rice, for example, over 400 QTLs have been reported for seed weight and shape traits (Huang et al., 2013). In linseed, in spite of the 44 reported QTLs/quantitative trait nucleotides (QTNs)/markers and underlying candidate genes, more light is certainly required for a better understanding of the genetic architecture of TSW.

GWAS has been a preferred strategy in recent times for genetic dissection of complex traits including seed weight/size in a number of crop plants such as wheat, soybean, rice, maize, and oilseed rape (Li et al., 2014; Lu et al., 2017; Kumar et al., 2020; Qi et al., 2020; Tao et al., 2020; Zhang et al., 2020; Chaurasia et al., 2021; Niu et al., 2021). In linseed, GWAS has been used for genetic dissection of flowering time traits, days to maturity, plant height, fiber-related traits, seed weight and size, pasmo resistance, mucilage content, and important agronomic traits (Soto-Cerda et al.,2018; Xie et al., 2018a; Xie et al., 2018b; He et al., 2019; Singh et al., 2019; Guo et al., 2020; Soto-Cerda et al., 2021; Saroha et al., 2022a; Yadav et al., 2022). For genotyping, reduced representation sequencing approach such as Genotyping by Sequencing (GBS) and SLAF has been successfully used for genotyping and further deployed in GWAS studies in different crop plants (Elshire et al., 2011; Hirsch et al., 2014; Kujur et al., 2015; Xie et al., 2018a; Xie et al., 2018b; Milner et al., 2019).

In present study, genetic dissection of TSW trait in linseed was undertaken using multi-locus GWAS methods in a panel of diverse germplasm accessions conserved in Indian National Genebank (INGB) to identify quantitative traits nucleotides (QTNs) and associated candidate genes.

## Material and methods

### Plant materials, field evaluation, and statistical analysis

Association mapping panel of 131 germplasm accessions (**Supplementary Table S1**) from a total of 220 accessions grown in five environments in three consecutive years (Saroha et al., 2022a; Saroha et al., 2022b) were used in this study. Observations on TSW were recorded on three representative plants from each accession in all five environments at two locations including Delhi (28°38’53.7”N 77°09’05.4”E) for 3 years (2017-18: DL17-18; 2018-19: DL18-19 and 2019-20: DL19-20) and Akola (20°42’03.2”N 77°01’53.6”E) for 2 years (2018-19: AK18-19 and 2019-20: AK19-20). Data analysis for each individual environment was done and adjusted means were generated in “R” with the package augmentedRCBD version 0.1.5 (Aravind et al., 2021), and the homogeneity of variances was tested by Bartlett’s chi-square test. Adjusted means were used for the calculation of descriptive statistics, frequency distribution plots, and GWAS.

### Multi-locus genome-wide association study

SNP data on the association mapping panel (131 accessions) was previously generated using genotyping by sequencing approach (Saroha et al., 2022a). Total of 68,925 SNPs distributed on 15 chromosomes were employed for multi-locus genome-wide association study (ML-GWAS) for the five independent environments using mrMLM package version 4.0.2 (Wen et al., 2018) of “R”. All the six multi-locus models: mrMLM (Wang et al., 2016), FASTmrMLM (Tamba and Zhang, 2018), FASTmrEMMA (Wen et al., 2018), pKWmEB (Ren et al., 2018), pLARmEB (Zhang et al., 2017), and ISIS EM-BLASSO (Tamba et al., 2017) were deployed for analysis using default values for all the parameters. A threshold of logarithm of odds (LOD) score ≥ 3.0 was used to consider the QTNs statistically significant for the trait to balance the high-power and low false positive rate for QTN detection using ML-GWAS methods (Zhang et al., 2019). For eliminating the model and environment biasness, QTNs identified either by ≥ 2 models or in ≥ 2 environments were considered as stable QTNs.

### Identification of candidate genes and *in silico* gene expression study

Genes around 30 kb region (30 kb upstream and 30 kb downstream, total 60 kb) of the stable QTNs were considered as potential candidate genes. Annotated genes as per the flax genome annotation (You et al., 2018a; You and Cloutier, 2020) were aligned against the Swiss-Prot sequences (reviewed and manually curated) of the Plants taxonomic division at the Uniprot database (https://www.uniprot.org/). Functional annotation of the potential candidate genes was performed as per the best hit.

*In silico* gene expression of the candidate genes was studied using publicly available transcriptome data of the rice variety *Nipponbare* available at Rice Genome Annotation Project database (http://rice.uga.edu/index.shtml) at four developmental stages: Embryo - 25 Days After Pollination (DAP) (SRX100753), Endosperm - 25 DAP (SRX100754) and Seed - 5 DAP (SRX100749), and 10 DAP (SRX100755). Candidate genes were aligned against the *Nopponbare* protein sequences and heatmap plots of the best hit were generated by the ComplexHeatmap package version 2.10.0 (Gu et al., 2016) of “R”.

## Results

### Phenotypic variation

**Figure 1:**
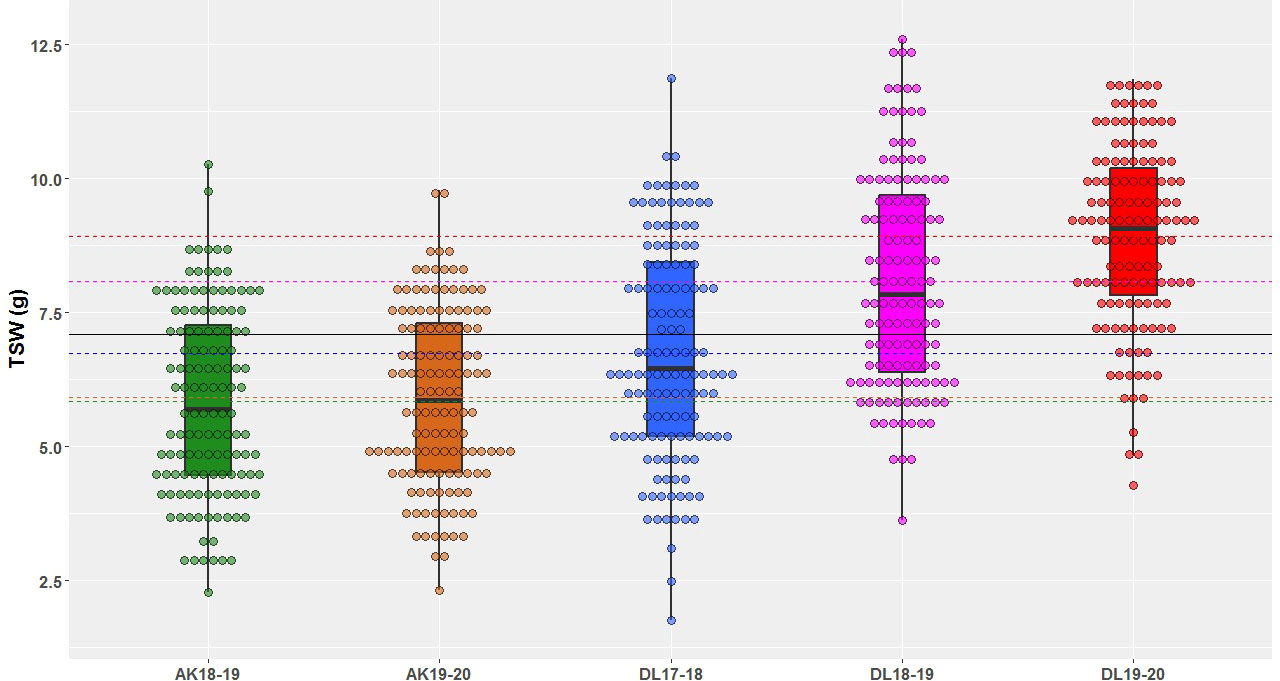
Thousand-seed weight phenotypic variation illustrated in box & dot plots in association mapping panel of linseed germplasm accessions grown at Akola during years 2018-19 (AK18-19) and 2019-20 (AK19-20) and Delhi during 2017-18 (DL17-18), 2018-19 (DL18-19), and 2019-20 (DL19-20).

A wide range of phenotypic variation was observed for TSW in AM panel under all the five environments (**Table 1** and **Figure 1**). The heterogeneity of the error variances for TSW was high for the studied environments and, therefore, analysis was performed independently for each of the environments. Among all the five environments, the lowest and highest TSW value was 1.76 g and 12.52 g, respectively. The mean TSW was significantly lower at Akola location than at Delhi location as the former comes under Zone-3 of linseed growing areas of India and the later under Zone-2. At Akola, mean TSW for the year 2018-19 and 2019-20 was 5.85 g and 5.92 g with CV of 29.8 and 27.57, respectively. At the Delhi location for the year 2017-18, 2018-19, and 2019-20 mean TSW was 6.73, 8.09, and 8.93 with CV of 30.10, 24.86, and 18.94, respectively. The variation within each single environment was statistically significant as evident in the analysis of variance (ANOVA) (**Supplementary Table S2**).

**Table 1:** Descriptive statistics of thousand seed weight (TSW) in association mapping panel in five environments at Akola (AK) and Delhi (DL).

Environment	Min	Max	Mean	Stand. dev	Coeff. var	Std. error
AK18-19	2.28	10.27	5.85	1.74	29.80	0.15
AK19-20	2.31	9.78	5.92	1.63	27.57	0.14
DL17-18	1.76	11.88	6.73	2.02	30.10	0.18
DL18-19	3.62	12.52	8.09	2.01	24.86	0.18
DL19-20	4.27	11.85	8.93	1.69	18.94	0.15

### Genome-wide association study for thousand-seed weight

**Figure 2:**
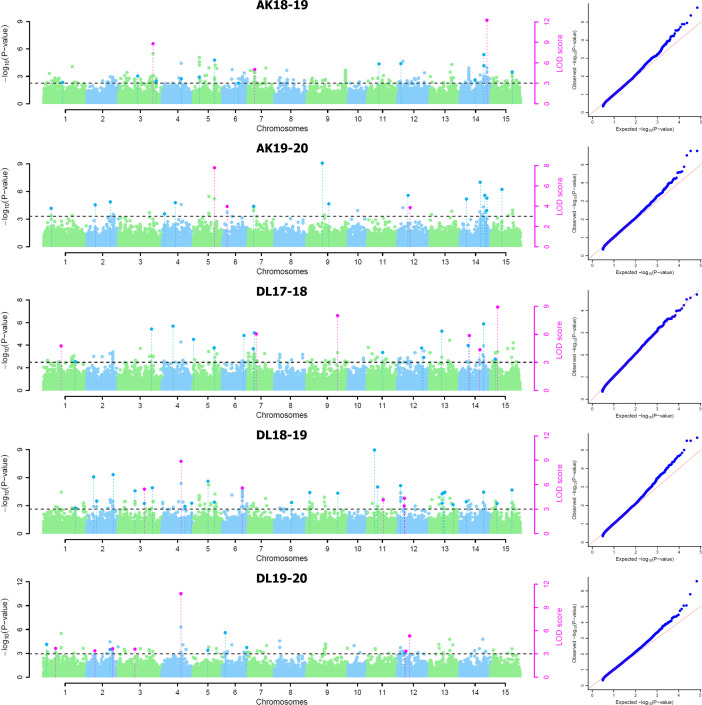
Combined Manhattan plots and respective quantile–quantile plots of five ML-GWAS methods for thousand-seed weight trait in five environments at Akola during 2018-19 (AK18-19) and 2019-20 (AK19-20) and New Delhi during 2017-18 (DL17-18), 2018-19 (DL18-19), and 2019-20 (DL19-20). LOD threshold score of 3.0 is indicated as dashed line in Manhattan plots. Dots above the threshold line represent significant QTNs, whereas QTNs identified in ≥ 2 methods are depicted in pink color.

For five environments, there were total 137 statistically significant QTNs identified with a threshold LOD score of ≥ 3.0 using five of the six ML-GWAS methods, of which 84 QTNs were unique across methods and environments (**Table 2**). Among the six ML-GWAS methods across the five environments, pLARmEB identified maximum of 39 unique QTNs followed by 27 QTNs each by mrMLM and ISIS EM-BLASSO. FASTmrMLM identified 24 unique QTNs, whereas the least number of seven QTNs were identified by FASTmrEMMA. There was no significant QTN identified by pKWmEB in any of the environments for TSW. With respect to the five environments, across five methods maximum unique QTNs were identified for Delhi 2018-19, followed by Delhi 2017-18 and Akola 2019-20. The five ML-GWAS methods combined Manhattan and Quantile-Quantile (QQ) plots for TSW trait for five environments depicting the significant QTNs have been shown (**Figure 2**).

**Table 2:** Distribution of QTNs identified for TSW in linseed across five environments and methods.

	AK18-19	AK19-20	DL17-18	DL18-19	DL19-20	TOTAL
mrMLM	2	6	8	10	5	31 (27)
FASTmrMLM	6	4	4	8	6	28 (24)
FASTmrEMMA	1	0	0	5	1	7 (7)
pLARmEB	5	8	8	12	10	43 (39)
ISIS EM-BLASSO	6	3	9	7	3	28 (27)
TOTAL	20 (16)	21 (18)	29 (21)	42 (32)	25 (13)	137 (84)

The number of QTNs in the parentheses are unique QTNs in respective environment/model.

**Figure 3:**
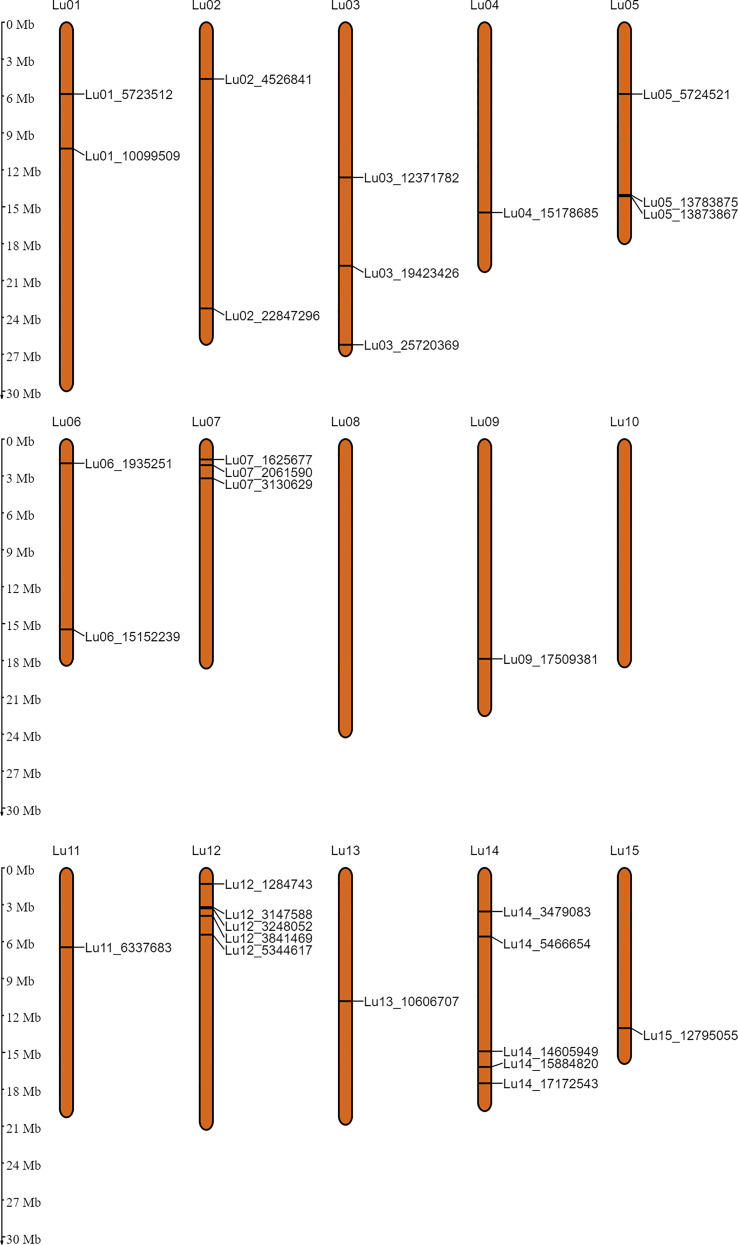
Genomic positions of the stable QTNs for thousand-seed weight on 15 chromosomes of linseed.

In order to identify the stable QTNs by removing method and/or environment biasness, QTNs identified by ≥ 2 models or in ≥ 2 environments were designated as stable QTNs. Accordingly, 30 stable QTNs have been identified for TSW (**Table 3**), and the rest were considered as potential QTNs (**Supplementary Table S3**). The LOD score and –log_10_(*p*) value for the stable QTNs ranged from 3.02 to 12.57 and 3.72 to 13.56, respectively, which explained up to 38.65% of the variation in the TSW trait across environments (**Table 3**). The genomic locations of all the identified stable QTNs for the TSW trait have been depicted on 15 chromosomes of linseed (**Figure 3**).

**Table 3:** List of stable QTNs identified for TSW in linseed.

QTN	Allele	Chromosome	Position (bp)	LOD score	-log_10_(*p*)	*r* ^2^ (%)	MAF	Environment (Method*)
*Lu01_5723512*	T/G	Lu01	5723512	3.48 - 4.60	4.20 - 5.38	2.84 - 5.89	0.43	DL19-20 (1,2,4)
*Lu01_10099509*	G/A	Lu01	10099509	4.61 - 4.92	5.39 - 5.71	7.40 - 9.53	0.23	DL17-18 (1,2)
*Lu02_4526841*	A/G	Lu02	4526841	3.22 - 3.58	3.93 - 4.31	4.69 - 5.90	0.27	DL19-20 (2,5)
*Lu02_22847296*	T/C	Lu02	22847296	3.44 - 4.38	4.16 - 5.15	4.41 - 7.12	0.32	DL19-20 (1,2,4,5)
*Lu03_12371782*	T/C	Lu03	12371782	3.02 - 5.25	3.72 - 6.05	3.32 - 7.50	0.48	DL18-19 (2); DL19-20 (2,4)
***Lu03_19423426***	**A/G**	**Lu03**	**19423426**	**3.88 - 7.04**	**4.62 - 7.90**	**10.37 - 16.22**	**0.25**	DL18-19 (1,2)
***Lu03_25720369***	**A/T**	**Lu03**	**25720369**	**8.74 - 8.84**	**9.65 - 9.75**	**23.03 - 25.55**	**0.39**	AK18-19 (1,3)
***Lu04_15178685***	**T/G**	**Lu04**	**15178685**	**3.66 - 11.90**	**4.40 - 12.88**	**14.84 - 38.65**	**0.25**	AK18-19 (5); DL18-19 (1,2,4,5); DL19-20 (1,2,4)
*Lu05_5724521*	C/A	Lu05	5724521	3.47 - 6.43	4.20 - 7.28	4.12 - 6.37	0.34	DL18-19 (4); DL19-20 (4)
***Lu05_13783875***	**G/C**	**Lu05**	**13783875**	**4.57 - 10.60**	**5.35 - 11.55**	**5.90 - 23.95**	**0.21**	AK19-20 (2,4); DL17-18 (5)
*Lu05_13873867*	G/A	Lu05	13873867	3.85 - 6.38	4.59 - 7.23	2.00 - 6.96	0.33	AK18-19 (4); DL18-19 (1)
***Lu06_1935251***	**A/G**	**Lu06**	**1935251**	**3.57 - 4.35**	**4.30 - 5.12**	**8.44 - 18.66**	**0.23**	AK19-20 (4,5)
*Lu06_15152239*	A/G	Lu06	15152239	5.19 - 7.03	5.99 - 7.89	3.09 - 9.37	0.32	DL18-19 (1,4,5)
*Lu07_1625677*	A/G	Lu07	1625677	3.98 - 4.46	4.73 - 5.23	3.73 - 3.82	0.44	AK19-20 (1); DL17-18 (1)
** *Lu07_2061590* **	**C/G**	**Lu07**	**2061590**	**4.98 - 6.16**	**5.77 - 7.00**	**6.38 - 14.17**	**0.48**	AK18-19 (1,2); DL17-18 (5)
***Lu07_3130629***	**A/G**	**Lu07**	**3130629**	**4.82 - 7.27**	**5.61 - 8.14**	**9.44 - 10.43**	**0.22**	DL17-18 (1,4)
***Lu09_17509381***	**G/T**	**Lu09**	**17509381**	**4.96 - 8.72**	**5.75 - 9.63**	**6.83 - 19.75**	**0.36**	DL17-18 (1,2,4); DL18-19 (1)
***Lu11_6337683***	**A/G**	**Lu11**	**6337683**	**3.41 - 4.84**	**4.13 - 5.63**	**4.04 - 11.84**	**0.44**	DL18-19 (3,5)
*Lu12_1284743*	A/G	Lu12	1284743	5.86 - 5.89	6.69 - 6.72	7.67 - 8.87	0.41	AK18-19 (2); DL18-19 (3)
*Lu12_3147588*	T/G	Lu12	3147588	3.28 - 3.87	3.99 - 4.62	1.06 - 3.20	0.46	DL18-19 (2,3,5)
*Lu12_3248052*	T/A	Lu12	3248052	3.08 - 4.81	3.79 - 5.60	1.77 - 2.80	0.35	DL18-19 (1,2); DL19-20 (4)
*Lu12_3841469*	G/A	Lu12	3841469	3.32 - 3.36	4.04 - 4.08	3.15 - 7.19	0.47	DL19-20 (1,4)
** *Lu12_5344617* **	**G/T**	**Lu12**	**5344617**	**3.69 - 8.45**	**4.43 - 9.35**	**8.29 - 13.65**	**0.23**	AK19-20 (1,2); DL19-20 (1,2,4)
*Lu13_10606707*	G/A	Lu13	10606707	3.73 - 6.35	4.46 - 7.19	3.56 - 7.67	0.48	DL17-18 (5); DL18-19 (4)
*Lu14_3479083*	A/G	Lu14	3479083	3.91 - 4.69	4.66 - 5.48	4.51 - 7.02	0.29	AK19-20 (4); DL18-19 (4)
***Lu14_5466654***	**T/A**	**Lu14**	**5466654**	**3.60 - 8.14**	**4.33 - 9.04**	**14.17 - 14.66**	**0.26**	DL17-18 (2,4)
*Lu14_14605949*	T/A	Lu14	14605949	3.82 - 4.76	4.56 - 5.54	4.09 - 6.83	0.46	DL17-18 (1,2,4)
*Lu14_15884820*	G/T	Lu14	15884820	5.08 - 7.19	5.88 - 8.06	3.91 - 9.15	0.40	AK18-19 (4); DL18-19 (4)
***Lu14_17172543***	T/C	**Lu14**	**17172543**	**9.41 - 12.57**	**10.34 - 13.56**	**18.04 - 25.98**	**0.35**	AK18-19 (2,4,5)
*Lu15_12795055*	A/G	Lu15	12795055	4.65 - 5.35	5.43 - 6.16	3.40 - 4.58	0.32	AK18-19 (2); DL18-19 (2)

*Methods: mrMLM (1); FASTmrMLM (2); FASTmrEMMA (3); pLARmEB (4) and ISIS EM-BLASSO (5).

Bold fonts indicate robust QTNs, accounting ≥ 10% phenotypic variation for the trait. Underlined QTNs are identified by ≥ 2 models as well as in ≥ 2 environments.

### Allelic effects of identified quantitative trait nucleotides on thousand-seed weight across environments

**Figure 4:**
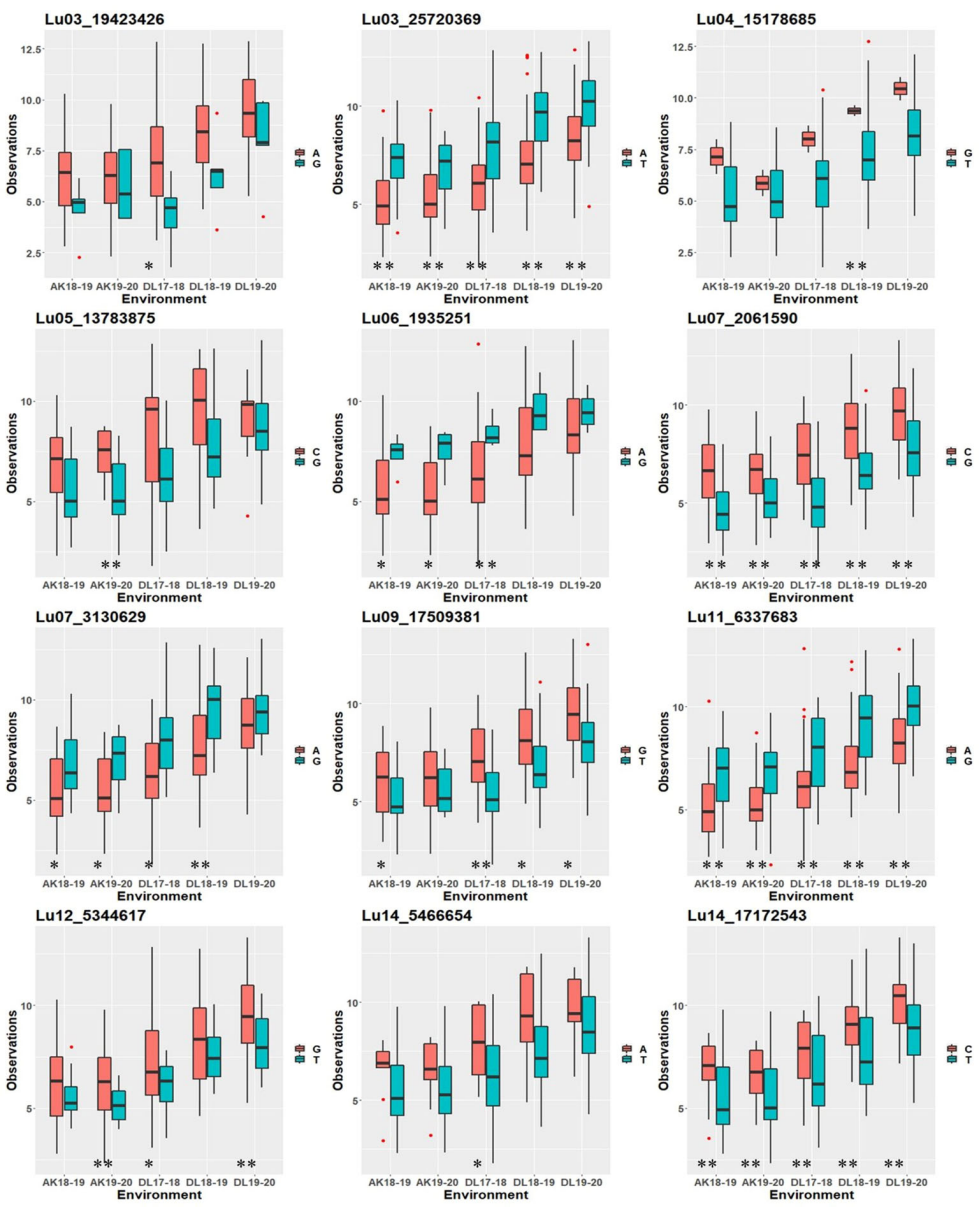
Box plot showing positive effect of alleles of the robust QTNs (*r* ^2^ ≥ 10.0%) on TSW trait values in five environments at Akola (2018-19 and 2019-20) and Delhi (2017-18, 2018-19, and 2019-20). Statistical significance at 0.01 and 0.05 were indicated with ** and *, respectively.

To study whether specific alleles of the QTNs carry any positive effect on the trait value in different environments, the robust QTNs having *r* ^2^ ≥ 10.0% in at least one of the identified environments/models were selected (**Table 3**). For each of the 12 selected robust QTN loci, the AM panel was divided into two groups based on the allele type they carried and the significance of the phenotypic difference between the two groups studied. For eight of the 12 studied QTNs, there was a statistically significant difference in TSW value between two groups carrying distinct alleles in at least three environments (**Figure 4**). For example, in the case of *Lu03_25720369*, accessions carrying allele “*Thymine*” *(T)* showed a higher five environment mean TSW 8.21 g compare with the accession group with another allele “*Adenine*” *(A)* having mean TSW 6.46 g. Four QTNs, *Lu03_25720369*, *Lu07_2061590*, *Lu11_6337683*, and *Lu14_17172543* showed a positive effect of the respective alleles on TSW consistent across the five environments.

### Identification of candidate genes for thousand-seed weight

Genes around the 30 kb region (30 kb upstream and downstream, total 60 kb) of the stable QTNs were extracted as per the linseed genome assembly (You et al., 2018a; You and Cloutier, 2020). A total of 333 genes were found around the stable QTNs. The list of the potential candidate genes for 30 stable QTNs of TSW and their functional annotation has been provided (**Supplementary Table S4**). Based on the functional annotation, 23 genes were shortlisted as candidate genes for TSW trait. Among the notable candidate genes included were *Lus10012572* and *Lus10012573* (*B3 domain-containing transcription factor*, *FUS3*), *Lus10015274* (*Shaggy-related protein kinase/Protein BRASSINOSTEROID INSENSITIVE 2*), *Lus10017640* and *Lus10017641* (*Auxin response factor 6*), *Lus10012582 *(*Cytochrome P450 84A/Ferulate-5-hydroxylase*), *Lus10030685* (*ANTIAUXIN-RESISTANT 3*), *Lus10030697* (*Probable ubiquitin conjugation factor E4/RING-type E3 ubiquitin transferase E4*), *Lus10037500* (*Protein pleiotropic regulatory locus 1*), *Lus10003792* (*E3 ubiquitin-protein ligase UPL5/Ubiquitin-protein ligase 5*), *Lus10042538* (*WRKY transcription factor*), *Lus10015277* (*MALE DISCOVERER 1/LRR receptor-like serine/threonine-protein kinase*), *Lus10015279* (*Non-specific lipid-transfer protein, NLTP*), *Lus10023553* (*Glucose-1-phosphate adenylyltransferase*), *Lus10028804* (*SUMO-activating enzyme*), *Lus10033912* (*Protein SCARECROW*), *Lus10037503* and *Lus10037504* (*Sucrose nonfermenting 4-LIKE protein/CBS domain-containing protein CBSCBS3*) (**Table 4**).

**Table 4:** List of candidate genes for TSW in linseed.

Lu Gene ID	Best Uniprot hit	Gene name	Protein name	Cross-reference (KEGG)	Gene ontology (biological process)
*Lus10012572*	FUS3_ARATH	*FUS3; At3g26790; MDJ14.4*	B3 domain-containing transcription factor FUS3	ath:AT3G26790	Embryo development ending in seed dormancy; regulation of seed number and size
*Lus10012573*	FUS3_ARATH	*FUS3; At3g26790; MDJ14.4*	B3 domain-containing transcription factor FUS3	ath:AT3G26790	Embryo development ending in seed dormancy; regulation of seed number and size
*Lus10014382*	GLGB1_PEA	*SBEI*	Starch branching enzyme I		Starch biosynthetic process
*Lus10015279*	NLTP2_GOSHI		Non-specific lipid-transfer protein		Lipid transport; oil accumulation in seeds
*Lus10015956*	ACR4L_ARATH	*ACR4; At3g59420; F25L23.280*	Serine/threonine-protein Kinase-LIKE protein	ath:AT3G59420	Embryo development ending in seed dormancy
*Lus10023553*	GLGL1_SOLTU	*AGPS1*	Glucose-1-phosphate adenylyltransferase		Glycogen biosynthetic process; starch biosynthetic process
*Lus10028801*	SHL_ARATH	*SHL; SHL1; At4g39100; F19H22.200*	Chromatin remodeling protein (Protein SHORT LIFE)	ath:AT4G39100	Flower development; post-embryonic development; regulation of photoperiodism
*Lus10028804*	SAE2_ARATH	*SAE2; EMB2764; At2g21470; F3K23.23*	SUMO-activating enzyme subunit 2	ath:AT2G21470	Embryo development ending in seed dormancy
*Lus10028811*	SPD1_ARATH	*SPD1; At3g10420; F13M14.30*	Protein SEEDLING PLASTID DEVELOPMENT 1	ath:AT3G10420	Seed maturation
*Lus10033912*	SCR_ARATH	*SCR; SGR1; At3g54220*	Protein SCARECROW	ath:AT3G54220	Repressor of seed maturation
*Lus10037503*	SNF4_ARATH	*CBSCBS3; At1g09020;*	Sucrose nonfermenting 4-LIKE protein (CBS domain-containing protein CBSCBS3	ath:AT1G09020	Seed development
*Lus10037504*	SNF4_ARATH	*SNF4; CBSCBS3; At1g09020; F7G19.11*	Sucrose nonfermenting 4-LIKE protein (CBS domain-containing protein CBSCBS3)	ath:AT1G09020	Seed development
*Lus10012582*	C84A1_ARATH	*CYP84A1 FAH1 At4g36220 F23E13.110*	Cytochrome P450 84A (Ferulate-5-hydroxylase) (F5H)	ath:AT4G36220	Phenylpropanoid biosynthetic process; response to UV-B
*Lus10030685*	AAR3_ARATH	*AAR3 At3g28970 K5K13.8*	Protein ANTIAUXIN-RESISTANT 3	ath:AT3G28970	Regulates responses to auxin, ubiquitin E3 ligase complex-mediated proteolysis
*Lus10030687*	C93A1_SOYBN	*CYP93A1 Glyma03g29950*	3,9-dihydroxypterocarpan 6A-monooxygenase (Cytochrome P450 93A1)	gmx:100776878	Biosynthesis of the phytoalexin
*Lus10030697*	UBE4_ARATH	*PUB1 UFD2 At5g15400 T20K14_10*	Probable ubiquitin conjugation factor E4, (Plant U-box protein 1) (RING-type E3 ubiquitin transferase E4)	ath:AT5G15400	Protein ubiquitylation
*Lus10037500*	PRL1_ARATH	*PRL1 MAC2 At4g15900 dl3990w FCAALL.40*	Protein pleiotropic regulatory locus 1 (Protein PRL1) (MOS4-associated complex protein 2) (MAC protein 2)	ath:AT4G15900	Pleiotropic regulator of glucose, stress and hormone responses.
*Lus10017640*	ARFF_ARATH	*ARF6 At1g30330 T4K22.6*	Auxin response factor 6	ath:AT1G30330	Modulate early auxin response genes expression. Regulates stamen and gynoecium maturation.
*Lus10017641*	ARFF_ORYSJ	*ARF6 ARF6A Os02g0164900*	Auxin response factor 6 (OsARF6a)	osa:4328406	Auxin responsive gene expressions
*Lus10003792*	UPL5_ARATH	*UPL5 At4g12570 T1P17.160*	E3 ubiquitin-protein ligase UPL5 (Ubiquitin-protein ligase 5)	ath:AT4G12570	Leaf senescence through ubiquitination and subsequent degradation of WRKY53.
*Lus10042538*	WRK74_ARATH	*WRKY74 At5g28650 F4I4.30*	WRKY transcription factor 74	ath:AT5G28650	Elicitor-responsive *cis*-acting element and gene expression
*Lus10015274*	KSG7_ARATH	*DWF12 SK21 UCU1 At4g18710 F28A21.120*	Shaggy-related protein kinase eta (ASK-eta) (Protein BRASSINOSTEROID INSENSITIVE 2) (Shaggy-related protein kinase 21)	ath:AT4G18710	Negative regulator in brassinosteroid signal transduction pathway. Auxin signalling pathway. Stomatal development, cell division, MAPK signalling; Phosphorylation of WRKY46, WRKY54 and WRKY70
*Lus10015277*	MDIS1_ARATH	*MDIS1 At5g45840 K15I22.4*	Protein MALE DISCOVERER 1(Probable LRR receptor-like serine/threonine-protein kinase)		Involved in the pollen tube perception of the female signal.
*Lus10010348*	VQ9_ARATH	*VQ9 At1g78310 F3F9.15*	VQ motif-containing protein 9 (AtVQ9)	ath:AT1G78310	Functions as a negative regulator of salt stress response. Functions as repressor of WRKY under salt stress.

### *In silico* expression of the candidate genes

**Figure 5:**
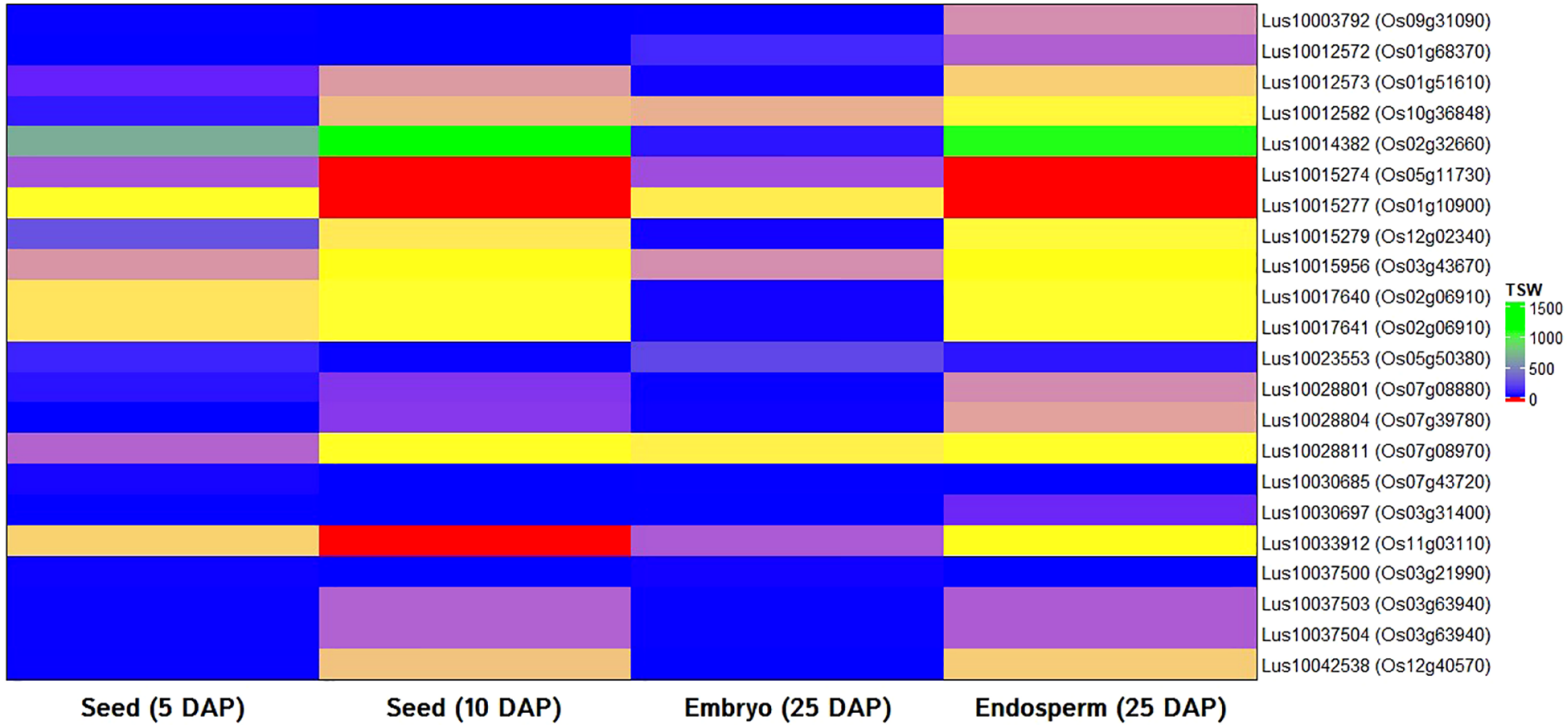
*In silico* gene expression of orthologs of candidate genes for thousand-seed weight in linseed using RNA-seq data of rice seeds (5 and 10 days after pollination: 5 DAP; 10 DAP), embryo (25 DAP), and endosperms (25 DAP). Transcriptome data of the rice variety *Nipponbare* available at Rice Genome Annotation Project database was used.

In order to validate the identified candidate genes and have the supporting evidence of their possible function in seed weight trait in model plants, *in silico* expression analysis was performed with rice ortholog of selected candidate genes using rice RNA-seq data as a basis. All but one (*Lus10010348*) candidate genes showed significant hit and found ortholog in the rice genome. For the expression study, rice RNA-seq data of seeds at 5 and 10 DAP, the embryo (25 DAP), and endosperm (25 DAP) tissues were used. All 22 genes showed good expression in either developing seeds, embryo or endosperm tissues (**Figure 5**).

## Discussion

### Variation in thousand-seed weight trait in linseed

Substantial variation for TSW trait was observed in the AM panel for all the studied environments (**Figure 1** and **Table 1**). Owing to the high-error variance (environmental factor), especially across geographical areas, statistical analyses were performed independently for each individual environment. GWAS studies performed in linseed for important agronomic and flowering time traits also followed a similar approach (Xie et al., 2018b; Soto-Cerda et al., 2021). There was a significant difference in the trait expression between the two geographical locations. Linseed accessions displayed relatively lower seed weight in Akola compared with Delhi. Akola and Delhi fall under the 2^nd^ and 3^rd^ linseed growing zones in India and have significant environmental/temperature differences due to latitudinal locations and thus have shorter growing seasons at the former location. The differences in trait expression at the two geographical locations were also expected, as the higher temperature is known to negatively affect plant growth and development and, thereby, the seed yields (Li et al., 2014; Čeh et al., 2020). Significant location effect on TSW trait in linseed was also observed in GWAS studies using Canadian flax core collection (Soto-Cerda et al., 2014).

### Favorable alleles of robust quantitative trait nucleotides for thousand-seed weight

The positive effect of alleles of 12 robust QTNs on TSW was observed in this study (**Figure 4**). Alleles with a positive effect on the trait would be helpful for trait improvement, as it would facilitate combining positive alleles in the genetic background of popular varieties (Zhang et al., 2012). These alleles would also help in the selection of parents for pyramiding in the desired genetic background. Favorable SSR alleles for TSW were reported earlier in Canadian flax core collection (Soto-Cerda et al., 2014). Combining the positive alleles of important yield components would make a pertinent strategy for yield improvement through its components (Shi et al., 2009; Soto-Cerda et al., 2014).

### Comparison of identified QTNs with earlier known QTLs/QTNs/markers

For TSW, a total of 44 QTLs have been identified so far from the earlier studies (Soto-Cerda et al., 2014; Wu et al., 2018; Xie et al., 2018a; Xie et al., 2018b, Guo et al., 2020, You and Cloutier, 2020). The 30 QTNs identified in this study were checked for co-location with earlier known QTLs/markers for TSW in linseed.

It was observed that a few of the stable QTNs were in close physical proximity of at least five earlier known markers associated with TSW on the linseed pseudomolecules (You et al., 2018a; You and Cloutier, 2020). SSR markers *Lu58a* and *Lu2555* were among the first reported markers associated with TSW (Soto-Cerda et al., 2014). QTNs *Lu12_3841469*, *Lu12_3248052*, *Lu12_3147588*, *Lu12_5344617* and *Lu12_1284743* were 0.04, 0.55, 0.66, 1.54, and 2.52 Mb close, respectively, to *Lu58a* on chromosome 12 (You and Cloutier, 2020). QTN *Lu06_15152239* was co-located 0.89 Mb close to *Lu2555* on chromosome 6. The same QTN was located just 0.2 Mb away from marker “*scaffold1491_58878*” for TSW (Xie et al., 2018a; You and Cloutier, 2020). Another QTN *Lu02_22847296* was also found in the proximity of 0.44 and 0.86 Mb to markers “*scaffold43-1111162*” and “*scaffold107-300735*”, respectively on chromosome 2 (You and Cloutier, 2020). Co-locating these QTLs in the present work and other independent studies (Soto-Cerda et al., 2014; Xie et al., 2018a) highlights their importance in the regulation of TSW in linseed. This also underpins the power of ML-GWAS methods in the genetic dissection of complex traits. The QTNs/markers identified for TSW in this study as well as the earlier identified QTNs/markers for TSW, oil and seed yield (Soto-Cerda et al., 2014; Kumar et al., 2015; You et al., 2018b; You and Cloutier, 2020) have been depicted on linseed chromosomes (**Supplementary Figure S1**) to facilitate identification of QTL/QTN rich regions associated with these economically important traits.

In addition, the QTNs near the known QTLs/markers, other QTNs identified in this study are novel and hitherto unknown QTNs/genomic regions for TSW in linseed (**Table 3**).

### Candidate genes for TSW trait in linseed

“*Priori knowledge of biology*” is one of the most important corner stones in identifying candidate genes in GWAS studies (Burghardt et al., 2017). As per the LD Decay (Saroha et al., 2022a), 23 candidate genes have been identified from 30 kb regions (total 60 kb up- and downstream) around the stable QTNs. Earlier studies in linseed reported important candidate genes for seed weight including *leucine-rich receptor-like protein kinase*, *cytochrome P450 family protein*, *WRKY genes*, *GRAS family transcription factors*, *Pho1 (SPX & EXS domain-containing protein)*, *auxin canalization*, *kinase family*, *RING/U-box protein* and, a few genes related to ubiquitin proteosome pathway (Kumar et al., 2015; Xie et al., 2018a; Guo et al., 2020). Indeed, in the present work, candidate genes such as *cytochrome P450 proteins* (*Lus10012582*, *Lus10030687*), *WRKY (Lus10042538)*, *Probable LRR receptor-like serine/threonine-protein kinase (Lus10015277)*, *SCARECROW/GRAS family protein* (*Lus10033912*), *RING-type E3 ubiquitin transferase/ubiquitin conjugation factor E4* (*Lus10030697*), and *E3 ubiquitin-protein ligase* (*Lus10003792*) have been identified underpinning their importance in the regulation of thousand seed weight in linseed.

In other plants, involvement of cytochrome P450 proteins in seed weight and yield is known (Huang et al., 2013; Ayalew et al., 2022). The crucial roles of ubiquitin-proteasome pathways in regulating seed size have been shown in several recent studies (Xia et al., 2013; Kelley, 2018; Xu and Xue, 2019; Wang et al., 2020). In flaxseed, six of the 13 identified candidate genes through GWAS were related to the ubiquitin-proteasome pathway (Guo et al., 2020), which highlighted the important role of deubiquitination in seed size regulation. In congruence, the present study identified seven candidates/potential candidate genes encoding RING-type E3 ubiquitin transferase for TSW (**Table 4** and **Supplementary Table S4**), which further substantiates the possible involvement of deubiquitination/ubiquitination in seed weight regulation in linseed.

Two promising candidate genes *Lus10017640* and *Lus10017641* for TSW showed similarity to *Auxin response factor 6* of Arabidopsis and rice with putative function in modulating early auxin response genes expression (**Table 4**). Role of auxin as a key regulator of seed and seed weight development is well established (Cao et al., 2020). A number of genes involved in auxin biosynthesis, supply, transport, metabolism, and auxin–BR signaling have been identified to play crucial role in varied aspect of seed development in different plant species (Dharmasiri et al., 2005; Bernardi et al., 2012; Ishimaru et al., 2013; Liu et al., 2015a; Liu et al., 2015b; Hirano et al., 2017; Shi et al., 2019; Cao et al., 2020).

One of the most important candidates identified for TSW in this study is *shaggy-related protein kinase/protein BRASSINOSTEROID INSENSITIVE 2 (BIN2)* (*Lus10015274*). Role of brassinosteroid hormone in normal growth and development of plants as well as regulating seed size/weight is well known (Wu et al., 2016; Song, 2017; Xiong et al., 2021). In rice, for grain width, a major QTL (*GW5/qSW5/GSE5*) was identified harboring a domestication related gene *GW5*, which encodes a protein with two IQ calmodulin-binding motifs (Shomura et al., 2008; Weng et al., 2008; Duan et al., 2017; Liu et al., 2017; Song, 2017). GW5 was shown to impart a negative effect on grain width as its reduced and upregulated expression gave wider and narrower grains, respectively (Duan et al., 2017; Liu et al., 2017). GLYCOGEN SYNTHASE KINASE 2 (GSK2), an ortholog of the Arabidopsis GSK3/SHAGGY-like kinase BIN2 was identified as a viable interacting partner of GW5 (Liu et al., 2017; Song, 2017). GSK2 is known negative regulator of brassinosteroid signaling and inhibit stomatal development by phosphorylation mediated inhibition of MAPKK kinase, YODA, and the MAPK kinases MKK4 and MKK5 (Li and Nam, 2002; Kim et al., 2012; Khan et al., 2013). In Arabidopsis, SHAGGY-like kinase BIN2 also promote and restrict vital process of asymmetric cell division (ACD) by phosphorylating MAPK-signaling components and the downstream transcription factor SPEECHLESS (SPCH), respectively (Houbaert et al., 2018). Therefore, *Lus10015274*, *a shaggy-related protein kinase/protein BIN2* is a promising candidate gene for TSW in linseed.

*WRKY* transcription factor gene (*Lus10042538*) and *LRR receptor-like serine/threonine-protein kinase* (*Lus10015277*) are other important candidates identified for TSW (**Table 4**). Genes encoding both WRKY and LRR were also earlier identified as candidate gene for TSW in linseed (Kumar et al., 2015). In Arabidopsis, *WRKY* and *leucine-rich repeat (LRR) KINASE* family genes are known to regulate seed size (Luo et al., 2005; Gu et al., 2017). It is interesting to note that WRKY46, WRKY54, and WRKY70 were shown to exert positive roles in brassinosteroid-regulated plant growth and negative role in drought stress response. Shaggy-related protein kinase/protein BIN2 reportedly destabilizes WRKY46, WRKY54, and WRKY70 by phosphorylation (Chen et al., 2017). It is further astonishing to know that candidate gene (*Lus10010348*) encoding VQ motif-containing protein 9 has been identified for TSW in this study. AtVQ9 in Arabidopsis act as a negative regulator of salt stress and repressor of WRKY (Hu et al., 2013). Another promising candidate gene (*Lus10003792*) encodes E3 ubiquitin-protein ligase 5, whose gene ontology function showed regulation of leaf senescence through ubiquitination and subsequent degradation of WRKY53 (Miao and Zentgraf, 2010).

Candidate gene *Lus10033912* encoding GRAS family protein 20/SCARECROW was identified for TSW, whose Arabidopsis counterpart was shown to be involved in ACD, specification of quiescent center and maintenance of surrounding stem cells toward radial pattern formation in roots. The *GRAS* gene was also found essential for cell division, shoot gravitropism, and regulation of radial organization of shoot axial organs (Heidstra et al. 2004; Wysocka-Diller et al., 2000; Sabatini et al., 2003; Sena et al., 2004). Role of SCARECROW-LIKE 15 in repressing the seed maturation by interaction with HISTONE DEACETYLASE19 is known (Gao et al., 2015).

Among others, an important gene *Lus10015279* (*non-specific lipid-transfer protein, NLTP*) showed possible involvement in multiple seed development-related processes such as pollen development, pollen tube adhesion-growth, seed coat development, seed maturation, germination, and fruit ripening (Missaoui et al., 2022). NLTPs are also known to play role in biosynthesis and storage of lipids in seeds. In sesame, two members of the NLTP gene family, *SiLTPI.23* and *SiLTPI.28*, have been identified for high oil content in seeds (Song et al., 2021). Another important gene *Lus10014382* (*1,4-alpha-glucan-branching enzyme 1*, *chloroplastic/amyloplastic* or *Starch branching enzyme I*, *SBEI*) showed function in glycogen and starch biosynthesis. Absence of the *SBEI* enzyme caused reduced amylopectin and starch, while increase in sucrose in the developing seeds, which resulted in increased osmosis and thereby larger cell size. In maturing seeds, loss of water leads to the cell shrinking and wrinkled seed phenotype (Bhattacharyya et al., 1993).

Orthologs of genes *Lus10012572* and *Lus10012573* (*B3 domain–containing transcription factor FUS3*) have been reported to play diverse roles in plant life cycle, such as seed germination, dormancy, embryo formation, seed and fruit development, and seed maturation (Santos-Mendoza et al., 2008). In Arabidopsis, *FUS3* positively regulates seed filling by suppressing expression of *TRANSPARENT TESTA GLABRA1* (*TTG1*) gene. *TTG1* negatively regulates accumulation/synthesis of the seed storage reserves such as storage proteins and fatty acids during embryogenesis. *TTG1* also indirectly suppresses the expression of a group of genes that either act as master regulators of seed development or are involved in synthesis/modification of fatty acids during seed development (Chen et al., 2015). *FUS3*, together with the *ABI3*, *LEC1*, and *LEC2*, also regulates biosynthesis of fatty acids in Arabidopsis (Mu et al., 2008; Roscoe et al., 2015). Recently, Ahmad et al. (2022) reported that, in tomato, overexpression of *VvFUS3* gene resulted in a reduction in seed number and seed weight, however, with no effect on size of the fruits. Another gene *Lus10023553* (*Glucose-1-phosphate adenylyltransferase* or *ADP-glucose synthase* or *ADP-glucose pyrophosphorylase*, *AGPase*) is possibly involved in the biosynthesis of glycogen and starch. In oilseeds, accumulation of starch during seed development is temporary and almost absent in mature seeds. Suppression of *AGPase* activity in Camelina seeds significantly increased seed size and weight (Na et al., 2018).

To study the possible role of the identified candidate genes in seed development process, *in silico* gene expression of orthologs of 22 candidate genes were studied using RNA-seq data of different seed developmental stages and tissues of rice (**Figure 5**). Orthology and *in silico* expression of candidate genes have been considered important to extrapolate the underlying gene function from model to other plant species (Das et al., 2016). *In silico* gene expression approach has earlier been employed in identification and successful validation of candidate genes for important traits including seed weight, seed development, seed yield, flowering time, agronomic traits in other plants, and flaxseed as well (Ngu et al., 2014; Soto-Cerda et al., 2021; Ayalew et al., 2022; Raboanatahiry et al., 2022).

## Conclusion

Present study reports genetic dissection of TSW trait in linseed using ML-GWAS approach. The study identified 84 unique significant QTNs for TSW, of which 30 QTNs were designated as “stable” QTNs as have been identified in two or more methods/environments. The stable QTNs accounted up to 38.65% of the trait variation. Several important candidate genes have also been identified including *shaggy-related protein kinase/BRASSINOSTEROID INSENSITIVE 2 (BIN2), ANTIAUXIN-RESISTANT 3, SCARECROW, RING-type E3 ubiquitin transferase E4, Auxin response factor, and WRKY transcription factor*. The study not only reported novel QTNs and candidate genes but also collocated a few QTNs near to the established QTLs/markers for TSW in linseed. Unravelling QTNs/markers/candidate genes associated with TSW in independent studies underpin the importance of these genomic regions/QTLs in regulation of TSW. With more light shed on different possible attributes of TSW, this study gives a novel insight in the genetic architecture of complex TSW trait in this ancient crop.

## Data availability statement

The datasets used in this study can be found in online repositories. The names of the repository/repositories and accession number(s) can be found below: https://www.ncbi.nlm.nih.gov/genbank/, PRJNA706105.

## Author contributions

DW, VK, and AK conceptualized the project and designed experiments. AS, DP, SG, and SU have done the investigation, data recording, and curation. AS, DW, JA, and SR have done statistical and GWAS analyses. JA provided germplasm resources. DW provided resources. DW, VK, AK, and KS facilitated funding acquisition. AS and DW wrote the original draft. KS, AK, MS, and DW reviewed the manuscript. All authors contributed to the article and approved the submitted version.

## Supplementary material

Supplementary Figure 1 Chromosomal positions of QTNs/QTLs for TSW, oil, and seed yield based on earlier and present study. QTL/QTN/markers from earlier published work (Soto-Cerda et al., 2014; Kumar et al., 2015; You et al., 2018b; You and Cloutier, 2020) within 3.0 Mb proximality of QTNs identified in this study have been depicted on linseed chromosomes (You et al., 2018a; You et al., 2018b; You and Cloutier, 2020).
