# Supplementary figures and images for: Genetic dissection of thousand-seed weight in linseed (Linum usitatissimum L.) using multi-locus genome-wide association study
Source: Front Plant Sci. 2023 Jun 2;14:1166728. doi: 10.3389/fpls.2023.1166728 (PMC10272591; doi:10.3389/fpls.2023.1166728)

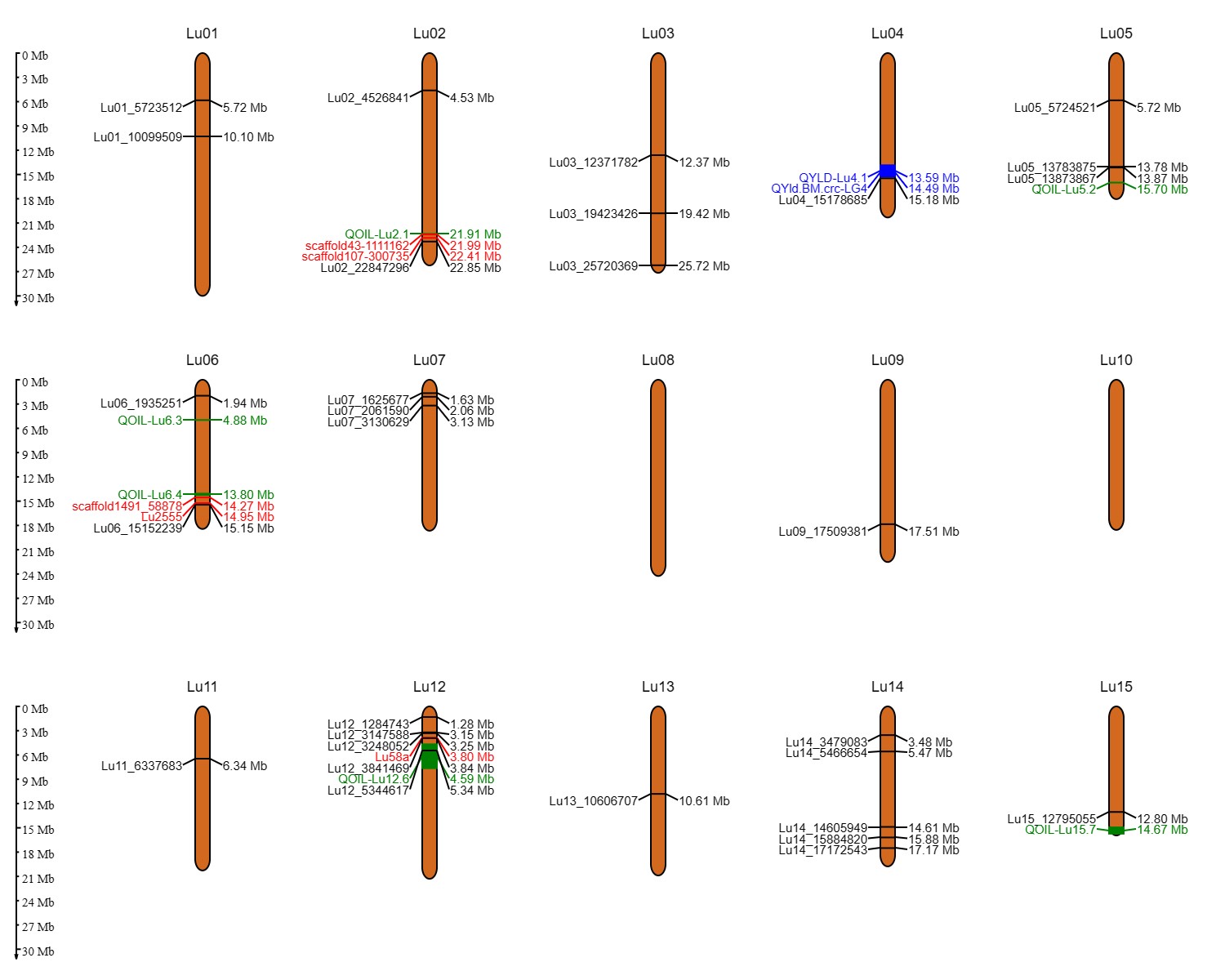

Supplement: Supplementary Figure 1 — Chromosomal positions of QTNs/QTLs for TSW, oil, and seed yield based on earlier and present study. QTL/QTN/markers from earlier published work (Soto-Cerda et al., 2014; Kumar et al., 2015; You et al., 2018b; You and Cloutier, 2020) within 3.0 Mb proximality of QTNs identified in this study have been depicted on linseed chromosomes (You et al., 2018a; You et al., 2018b; You and Cloutier, 2020). [file Image_1.jpeg]
