# Supplementary material for: Genetic dissection of thousand-seed weight in linseed (Linum usitatissimum L.) using multi-locus genome-wide association study
Source: Front Plant Sci. 2023 Jun 2;14:1166728. doi: 10.3389/fpls.2023.1166728 (PMC10272591; doi:10.3389/fpls.2023.1166728)
Supplement: Supplementary file 3 [file Table_2.docx]

**Supplementary table 2: Analysis of Variance for TSW trait in five environments.**

| **Source** | **Df** | **AK18-19** | **AK19-20** | **DL17-18** | **DL18-19** | **DL19-20** |
| --- | --- | --- | --- | --- | --- | --- |
| Block (ignoring Treatments) | 5 | 11.36 ** | 13.83 ** | 12.87 ** | 12.74 ** | 16.9 ** |
| Treatment (eliminating Blocks) | 222 | 2.5 ** | 2.04 ** | 3.27 ** | 3.57 ** | 2.67 ** |
| Treatment: Check | 2 | 10.39 ** | 5.93 ** | 10.11 ** | 7.82 ** | 6.68 ** |
| Treatment: Test and Test vs. Check | 220 | 2.43 ** | 2.01 ** | 3.21 ** | 3.53 ** | 2.63 ** |
| Residuals | 28 | 0.48 | 0.47 | 0.64 | 0.33 | 0.93 |

ns P > 0.05; * P ≤ 0.05; ** P ≤ 0.01
